# Association between anxiety and eating behaviors in patients with obesity

**DOI:** 10.1016/j.obpill.2022.100021

**Published:** 2022-05-17

**Authors:** Lizeth Cifuentes, Alejandro Campos, Maria Laura Ricardo Silgado, Sydney Kelpin, Jessica Stutzman, Maria Daniela Hurtado, Karen Grothe, Donald D. Hensrud, Matthew M. Clark, Andres Acosta

**Affiliations:** a Precision Medicine for Obesity Program and Division of Gastroenterology and Hepatology, Department of Medicine, Mayo Clinic, Rochester, MN, USA; b Department of Psychiatry & Psychology, Mayo Clinic, Rochester, MN, USA; c Division of Endocrinology, Diabetes, Metabolism, and Nutrition, Department of Medicine, Mayo Clinic Health System, La Crosse, WI, USA; d Division of General Internal Medicine, Mayo Clinic, Rochester, MN, USA

**Keywords:** Obesity, Emotional eating, Uncontrolled eating

## Abstract

**Background:**

Given the link between eating behavior and obesity, it is critical to identify individuals who have eating behaviors which contribute to obesity etiology. This study aimed to investigate the potential relationship between symptoms of anxiety and eating behaviors in patients with obesity.

**Methods:**

This was a cross-sectional study analyzing baseline characteristics of 438 patients with obesity (BMI>30). Patients completed the Hospital Anxiety and Depression Scale (HADS) questionnaire, the Three-Factor Eating Questionnaire R21 (TFEQ-R21), and the Weight Efficacy Lifestyle Questionnaire (WEL). Pearson correlation coefficients were used to evaluate the association between questionnaires. Two-sample independent *t*-test were conducted to examine differences in the TFEQ-R21 and WEL between low and moderate to severe levels of symptoms of anxiety.

**Results:**

Anxiety scores (HADS-A) positively correlated with two factors of the TFEQ, emotional eating (r = 0.36) and uncontrolled eating (r = 0.27). The HADS-A score was negatively correlated with self-efficacy to resist eating in all five situational factors on the WEL (p < 0.01). Patients with symptoms of anxiety additionally showed higher mean scores for emotional eating and uncontrolled eating (p < 0.001, respectively),and lower levels of cognitive restraint (p = 0.04)) on the TFEQ-R21.

**Conclusion:**

Patients with obesity who reported having anxiety symptoms had lower self-confidence to manage their eating and more emotional eating than patients with low anxiety symptoms. Clearly more needs to be learned about symtoms of anxiety and eating behaviors.

## 1 Introduction

Obesity is a growing public health global epidemic that affects over 600 million adults worldwide. Obesity contributes to the development of multiple metabolic, mechanical, and mental health disorders associated with increased mortality. The dominant pathogenesis is the imbalance in the processes involved in energy homeostasis, with an energy intake greater than energy expenditure, leading to energy storage [1]. Several problematic eating behaviors (i.e., binge eating episodes, loss of control over eating) and mood (i.e. depression or stress) have shown an association with obesity [2].

Anxiety has repeatedly been associated with obesity and eating disorders. Anxiety has a complicated impact on food consumption and can increase [[3], [4], [5]] or decrease food intake [6]. Emotional eating is an inclination to eat in response to positive or negative emotions, with the foods chosen predominantly being energy-dense and appealing [7]. Emotional eating can result from various causes, such as using food to deal with negative emotions or confusing internal hunger and satiety with physiological changes connected with emotions. Understanding the extent to which anxiety affects problematic eating behaviors might give valuable insight into the genesis of obesity and potentially lead to new individualized approaches to obesity treatment.

The relationship between eating behaviors and anxiety has not been well studied in patients with obesity. To date, three domains of eating behavior (uncontrolled eating, cognitive restraint, and emotional eating) have been linked to anxiety in women between 40 and 65 years [8] and assessed in university students, mainly with healthy weight [9]. While both studies suggest a relationship between anxiety and the three elements that influence eating behavior, they were done primarily on overweight patients and only a small fraction of patients who had obesity. Another factor that has been shown to influence eating behavior is self-efficacy, which refers to a person's confidence in executing a specific task [10]. Previous research has demonstrated that self-efficacy improves throughout obesity treatment and is associated with greater weight loss [11,12].

Based on the literature to date we hypothesize that there is a correlation between anxiety and eating behavior and that patients with higher symptoms of anxiety have more emotional eating and lower self-efficacy in resisting eating than patients with obesity and low symptoms of anxiety. The purpose of this study was to investigate the relationship between symptoms of anxiety and all three components of eating behavior and the five eating self-efficacy situational factors of weight control in patients with obesity.

## 2 Methods

### 2.1 Study design and participants

A total of 438 adults with obesity (BMI >30 kg/m^2^) and controlled comorbidities or other diseases were prospectively enrolled as part of two clinical trials: NCT03374956 and NCT04073394. Participants were recruited from the general population by advertisement on clinical trials.gov. All study participants did not take any medications that may have altered gastrointestinal motility, appetite, or absorption within the last 6 months, including tricyclic antidepressants and atypical antidepressants (e.g., bupropion, mirtazapine, trazodone). Patients included in the study did not have eating disorders (i.e., anorexia nervosa, bulimia nervosa, binge-eating disorder, rumination disorder, or avoidant/restrictive food intake disorder). The Institutional Research Review Board (IRB) approved the study, and all participants gave written informed consent following a thorough explanation of the study details. The results reported here constitute the baseline measurements prior to any intervention.

### 2.2 Bodyweight and anthropometrics

Weight was measured without shoes to ±0.1 kg with an electronic scale calibrated daily with standard weights. The waist circumference was measured at the midpoint between the lower border of the rib cage and the iliac crest by using a flexible inch tape. Hip circumference was measured at the anterior superior iliac spine level, when this could be felt, otherwise at the broadest circumference below the waist using a flexible inch tape.

### 2.3 Measures

#### 2.3.1 Symptoms of anxiety and depression

The Hospital Anxiety and Depression Scale (HADS) questionnaire is a widely used questionnaire for detecting symptoms of anxiety and depressive disorders [13]. The HADS comprises 14 items, seven related to anxiety symptoms and seven related to depressive symptoms. Each item is coded from 0 to 3. The scores for anxiety and depression can therefore vary from 0 to 21, depending on the presence and severity of the symptoms. The interpretation of the obtained score is as follows: HADS-A< 7: no symptoms of anxiety and HADS-A ≥ 7: symptoms of anxiety; HADS-D< 7: no symptoms of depression and HADS-D ≥ 7: symptoms of depression [14].

#### 2.3.2 Eating behaviors

The Three-Factor Eating Questionnaire (TFEQ-R21) [15] and the Weight Efficacy Lifestyle Questionnaire (WEL) [16] are two validated questionnaires designed to assess eating and weight control behaviors. Participants completed the TFEQ-R21 and the WEL on their baseline assessment visit. The TFEQ-R21 is a 21-item instrument that measures three domains of eating behavior: cognitive restraint (CR), uncontrolled eating (UE), and emotional eating (EE) [15]. The first twenty items are rated on a 4-point likert scale, and item 21 is answered through an 8-point likert scale. Before calculating domain scores, items 1–16 were reverse coded, and item 21 was recorded as follows: 1–2 scores as 1; 3–4 as 2; 5–6 as 3; 7–8 as 4. Domain scores were then calculated as a mean of all items within each domain; hence, domain scores also ranged from 1 to 4 (CR [six items], UE [nine items], and EE [six items]), with higher scores being indicative of greater CR, UE, and EE.

The Weight Efficacy Lifestyle Questionnaire (WEL) [16] is a 20-item instrument that measures five situational factors. The 20 items were rated on a 0–9 scale, with a score of 0 indicating “not confident at all that I can resist the desire to eat”, and a score of 9 indicating “very confident that I can resist the desire to eat.” The five situational factors consist of: negative emotions, availability, social pressure, physical discomfort, and positive activities, often used to assess eating self-efficacy and were calculated as the mean of all items within each group [16,17].

### 2.4 Statistical analysis

This was a cross-sectional study. Statistical analysis was performed using JMP®, Version 14.3.0 (SAS Institute Inc., Cary, NC, 1989–2019). Categorical variables are presented as percentages, and all continuous data are summarized as mean and standard deviation (SD). We used descriptive statistics to show demographic, anthropometric, and behavioral parameters. Categorical data were analyzed using Fisher's exact test. Pearson correlation coefficients were used to evaluate the association between continuous variables. Two-sample independent *t*-test were conducted to test the association of study variables with anxiety levels. All tests were two-tailed, and a p-value <0.05 was considered statistically significant. See Strengthening the Reporting of Observational Studies in Epidemiology (STROBE) guidelines checklist in online Supporting Information.

## 3 Results

### 3.1 Participant characteristics

Table 1 indicates the demographics and anthropometrics of the 438 study participants. Most participants were women (77%) and White individuals (92%), with a mean (SD) age of 41.1 (11) years, with a mean BMI of 38.9 kg/m^2^ (7.2). Women did not have a different BMI when compared to men (38.9 kg/m^2^ vs. 38.8 kg/m^2^; p = 0.96). The sample included 37% patients with obesity class I, 29% patients with obesity class II, and 34% patients with obesity class III. A review of electronic medical records indiated that there were 174 patients (39%) diagnosed with anxiety and 174 patients (39%) with a diagnosis of depression. When comparing the prevalence by gender, anxiety (41% vs. 34%; p < 0.001) and depression (44% vs. 24%; p < 0.001) were higher among women compared to men. The use of selective serotonin reuptake inhibitors (SSRIs) was reported in 73 (16%) patients, with no difference by gender.

**Table 1:** Baseline characteristics of the study cohort.

	Total (n = 438)	Women (n = 339)	Men (n = 99)	p-value
Age, years	42.1 (11)	42.4 (10.9)	41.0 (11.4)	0.24
BMI, kg/m^2^	38.9 (7.2)	38.9 (7.2)	38.8 (7.4)	0.96
Waist/hip, ratio	0.90 (0.1)	0.90 (0.1)	0.99 (0.1)	<0.001
Obesity Class				0.81
Obesity Class I, %	37%	35.7%	39.4%	
Obesity Class II, %	29%	29.5%	27.3%	
Obesity Class III, %	34%	34.8%	33.3%	
Diagnosis of anxiety, n	174 (39%)	140 (41%)	34 (34%)	<0.001
Diagnosis of depression, n	174 (39%)	150 (44%)	24 (24%)	<0.001
Use of SSRIs, n	73 (16%)	56 (16%)	17 (17%)	0.74

**Abbreviations**: BMI, body mass index; SSRI, Selective serotonin reuptake inhibitors. Data are shown as mean and standard deviation.

### 3.2 Eating behavior and symptoms of anxiety

Table 2 shows that HADS-A scores positively correlated with two factors of the TFEQ-R21, emotional eating and uncontrolled eating, and negatively correlated with the five situational factors measured on the WEL. Cognitive restraint only showed a negative correlation with the waist/hip ratio. Positive activities measured on the WEL were also correlated to age.

**Table 2:** Correlations between eating behaviors with demographics, anthropometrics, and symptoms of anxiety.

	Age	BMI	Waist/Hip	HADS-A
TFEQ-R21 Cognitive restraint	−0.04	−0.11	−0.18∗∗	0.09
TFEQ-R21 Emotional Eating	0.08	0.11	−0.09	0.36∗∗∗
TFEQ-R21 Uncontrolled Eating	−0.06	0.06	0.02	0.27∗∗∗
WEL Negative Emotions	−0.09	0.02	−0.02	−0.24∗∗∗
WEL Availability	−0.08	0.09	−0.03	−0.17∗∗∗
WEL Social Pressure	−0.04	0.09	0.02	−0.18∗∗∗
WEL Physical Discomfort	−0.04	0.06	0.04	−0.16∗∗
WEL Positive Activities	−0.12∗	0.06	−0.01	−0.19∗∗∗

Data shown as Pearson correlation coefficients. ∗ ​= ​P ​< ​0.05; ∗∗ ​= ​P ​< ​0.01; ∗∗∗ ​= ​P ​< ​0.001.

**Abbreviations**: BMI, body mass index; HADS, Hospital Anxiety and Depression Scale; TFEQ, Three-Factor Eating Questionnaire; WEL, Weight Efficacy Lifestyle Questionnaire.

**Fig. 1:**
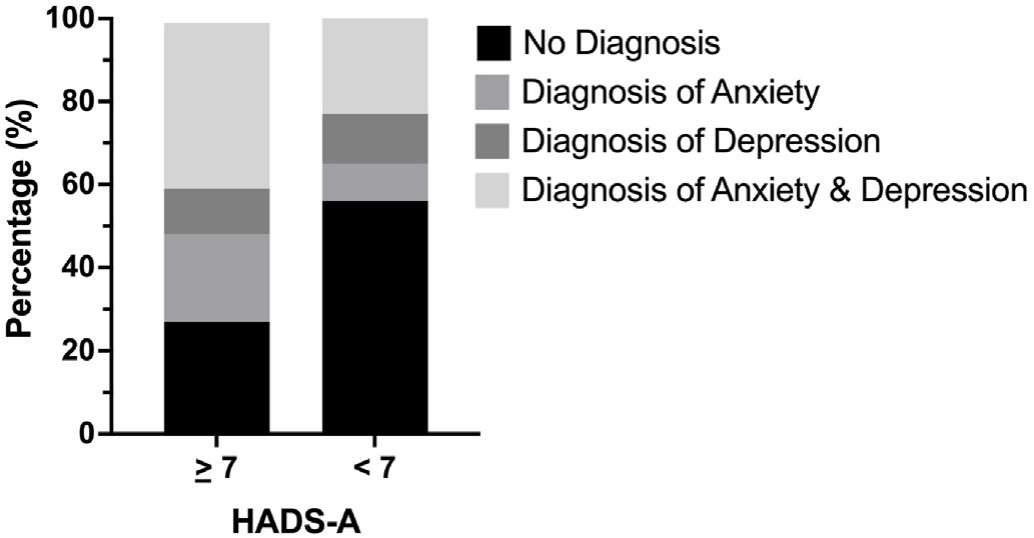
Diagnosis of anxiety and depression among patients with anxiety symptoms. Of 117 patients with HADS-A ≥7: 21% had a diagnosis of anxiety, 11% depression, and 40% had both. Of 312 patients with HADS-A ≤7: 9% had a diagnosis of anxiety, 12% depression, and 23% had both.

Based on the level of anxiety on the HADS-A, 117 patients had symptoms of anxiety (77% females, mean BMI of 39.3 kg/m^2^ [7.3]), while 321 patients had low or moderate symptoms of anxiety (77% females, mean BMI of 38.8 kg/m^2^ [7.2]). Eighty-eight patients (73%) with symptoms of anxiety had a diagnosis of anxiety, depression, or both. In contrast 142 patients (44%) with no symptoms of anxiety were diagnosed with anxiety, depression, or both (see Fig. 1). Of patients with symptoms of anxiety, 32 (13%) were prescribed an SSRI, compared to 41 (14%) patients without symptoms of anxiety.

**Fig. 2:**
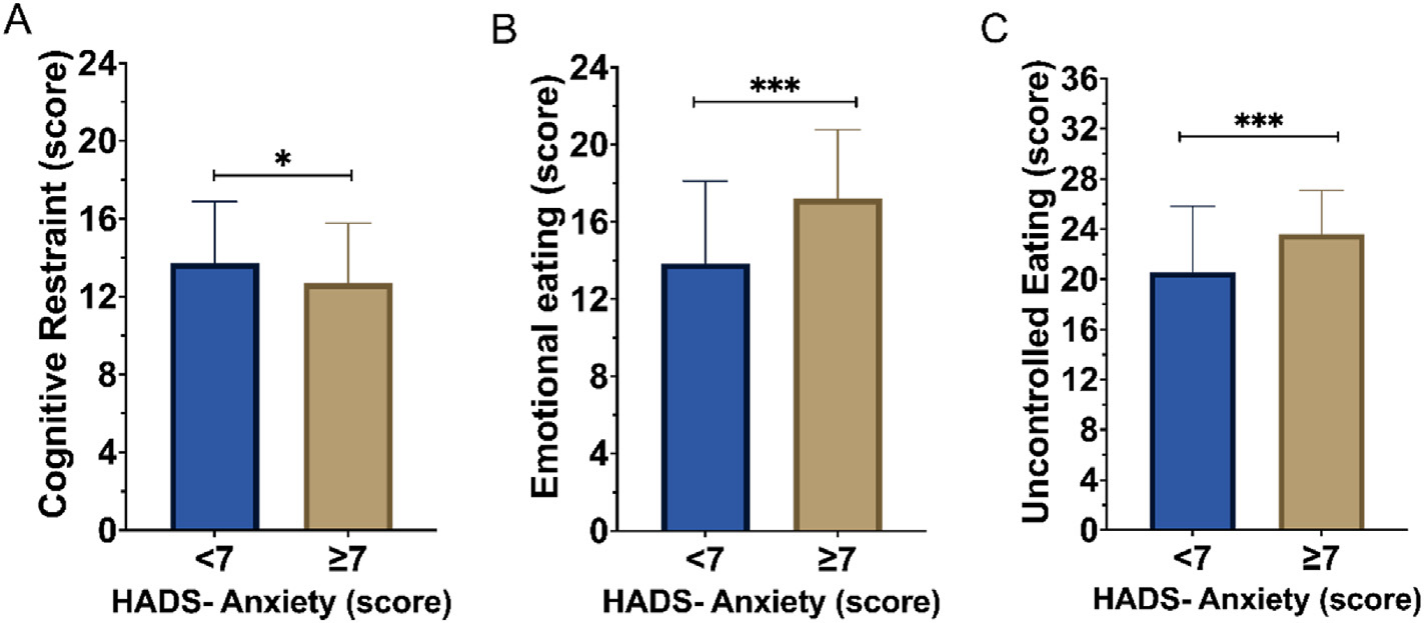
Anxiety symptoms levels and the three domains of eating behaviors by the Three-Factor-Eating-Questionnaire. A) Cognitive Restraint with 6 items compared between patients with and without symptoms of anxiety. B) Emotional Eating with 6 items between patients with and without symptoms of anxiety. C) Uncontrolled Eating with 9 items between those with and without symptoms of anxiety.

Table 3 summarizes the average scores in the HADS, TFEQ-21, and the WEL by classifying our cohort based on the symptoms of anxiety. Among the TFEQ-21 factors, cognitive restraint, emotional eating, and uncontrolled eating were significantly different between patients with no symptoms of anxiety and patients with symptoms of anxiety (p = 0.04; p < 0.001; and p < 0.001, respectively) (see Fig. 2). Regarding the five situational factors measured on the WEL, patients with no symptoms of anxiety had higher self-efficacy to resist eating for negative emotions, when food is available, social pressure, physical discomfort, and positive activities (p < 0.001, respectively). Patients with symptoms of anxiety were younger than patients with no symptoms of anxiety (40.1 [11.9] vs. 42.9 [11.9], p = 0.02). There were no differences in sex, BMI, or waist/hip ratio between the two groups (see Table 4).

**Table 3:** Symptoms of anxiety versus no symptoms of anxiety: depression and eating behaviors.

	No Anxiety Symptoms (<7) (n = 321)	Anxiety Symptoms (≥7) (n = 117)	Difference (CI)	p-value
HADS-A, points	2.9 (1.9)	8.9 (1.9)	−5.9 (−6.4–−5.5)	<0.001
HADS-D, points	2.7 (2.3)	4.9 (3.2)	−2.2 (−2.8–−1.5)	<0.001
TFEQ-R21 Cognitive restraint, points	13.7 (3.1)	12.7 (3.2)	1 (0.1–1.9)	0.04
TFEQ-R21 Emotional Eating, points	13.8 (4.2)	17.2 (3.5)	−3.4 (−4.5–−2.2)	<0.001
TFEQ-R21 Uncontrolled Eating, points	20.6 (5.2)	23.6 (3.5)	−3 (−4.3–−1.8)	<0.001
WEL Negative Emotions, points	21.1 (8.6)	16.4 (8.7)	4.6 (2.6–6.6)	<0.001
WEL Availability, points	17.7 (8.1)	14.7 (7.3)	2.9 (1.3–4.6)	<0.001
WEL Social Pressure, points	22.1 (8.5)	18.9 (7.8)	3.2 (1.4–4.9)	<0.001
WEL Physical Discomfort, points	25.7 (6.7)	22.8 (7)	2.9 (1.3–4.4)	<0.001
WEL Positive Activities, points	25.4 (7.0)	21.9 (7.7)	3.5 (1.8–5.1)	<0.001

**Abbreviations**: HADS, Hospital Anxiety and Depression Scale; TFEQ, Three-Factor Eating Questionnaire; WEL, Weight Efficacy Lifestyle Questionnaire. Data are shown as mean and standard deviation.

**Table 4:** Relationship between Anxiety Symptoms and demographics.

	No Anxiety Symptoms (<7) (n = 321)	Anxiety Symptoms (≥7) (n = 117)	Difference (CI)	p-value
Women, n	248 (77.3%)	91 (77.8%)	0% (−0.1 – 0.1	0.48
Age, years	42.9 (11.9)	40.1 (11.9)	2.8 (0.3–5.3)	0.02
BMI, kg/m^2^	38.8 (7.2)	39.3 (7.3)	−0.5 (−2.1–1.0)	0.49
Waist/hip, ratio	0.92 (0.1)	0.93 (0.01)	−0 (−0.02–0.01)	0.35
**Abbreviations**: BMI, body mass index. Data shown as mean and standard deviation.				

In adults with symptoms of anxiety, 33 patients (28%) additionally showed symptoms of depression. Table 5 summarizes the average scores in the HADS, TFEQ-21, and the WEL by classifying our cohort based on the depression symptoms. There were no differences in eating behaviors between patients with no symptoms of depression and patients with symptoms of depression.

**Table 5:** Co-morbid symptoms of depression versus no symptoms of depression: eating behaviors.

	No Depression Symptoms (<7) (n = 84)	Depression Symptoms (≥7) (n = 33)	Difference (CI)	p-value
HADS-A, points	8.6 (1.7)	9.4 (2.3)	−0.8 (−1.6–−0.1)	0.03
HADS-D, points	3.1 (1.8)	8.9 (1.9)	−5.8 (−6.7–−5.0)	<0.001
TFEQ-R21 Cognitive restraint, points	12.7 (3.0)	12.6 (3.3)	−0.1 (−8.4–8.1)	0.93
TFEQ-R21 Emotional Eating, points	17.1 (3.3)	17.8 (4.1)	−0.7 (−7.5–9.2)	0.80
TFEQ-R21 Uncontrolled Eating, points	23.6 (3.4)	23.3 (3.7)	−0.3 (−8.7–7.1)	0.94
WEL Negative Emotions, points	16.3 (7.9)	16.3 (10.2)	0.08 (−5.3–5.4)	0.97
WEL Availability, points	14.1 (7.1)	16.1 (7.6)	1.9 (−3.3–7.6)	0.46
WEL Social Pressure, points	18.7 (7.4)	19.3 (8.6)	0.6 (−4.2–5.9)	0.82
WEL Physical Discomfort, points	22.7 (6.4)	22.9 (8.4)	0.2 (−5.2–5.5)	0.94
WEL Positive Activities, points	22.2 (7.1)	21.3 (8.6)	−0.9 (−6.7–4.1)	0.73

**Abbreviations**: HADS, Hospital Anxiety and Depression Scale; TFEQ, Three-Factor Eating Questionnaire; WEL, Weight Efficacy Lifestyle Questionnaire. Data are shown as mean and standard deviation.

## 4 Discussion

In this study of a large sample of patients with obesity seeking obesity treatment, symptoms of anxiety at the time of enrollment into treatment was associated with problematic eating behaviors. Specifically, adults with obesity who endorsed having symptoms of anxiety reported lower cognitive restraint, more emotional eating, more uncontrolled eating, and lower eating self-efficacy in all five unique situational factors. Furthermore, when examining the impact of symptoms of anxiety on eating behaviors, these increased as anxiety levels increased. If confirmed by other investigators with more diverse samples, the results can potentially contribute to implementing a tailored approach to obesity treatment.

Obesity is associated with anxiety disorders in adults in several ways. The strongest association has been between severe obesity (BMI ≥35 kg/m2) and anxiety symptoms [18]. Patients with obesity have reported lower levels of social support and may face weight discrimination compared to healthy-weight persons, which adds to psychological discomfort and consequent anxiety [19]. Also, eating preoccupation is more prevalent among people with obesity and leads to anxiety [20]. Patients with obesity also reported decreased levels of physical activity, which is associated with higher anxiety and depressive symptoms [21]. In contrast, stress-induced eating may be one factor contributing to the development of obesity. High anxiety levels relate to an overall increase in food intake, but specifically with an increase in high-fat and high-carbohydrate meals [3,22,23]. In addition, chronic stress levels can be associated with appetite dysregulation and weight gain by a dysregulation in the hypothalamic-pituitary-adrenal axis [24].

Obesity and depression have been linked in several studies. In our study, based on review of electronic medical records, 174 (39%) of our patients had been diagnosed with anxiety. Studies have reported that patients with obesity have a 55% greater chance of being diagnosed with depression, whereas patients with depression have a 58% increased risk of having obesity [25]. Higher depression symptoms are linked to emotional eating with a dose-response association [26]. In addition, eating behaviors can indirectly impact depression by establishing hazardous dietary patterns and obesity, both of which have been linked to an increased risk of depression in the future [27,28]. Participants with symptoms of anxiety in our study also reported greater depressive symptoms, however, the co-morbid symptoms of anxiety and depression did not have an additive effect on their eating behaviors.

People may use a range of coping methods when in anxiety-producing situations to help decrease their feelings of unease [29]. Negative emotions, such as anxiety, have the potential to influence meal selections toward energy-dense “comfort meals,” such as sweets and chocolate [24,29]. This may be a learned behavior owing to food symbolism, in which sweet foods represent affection and caring, and eating these foods is a means of coping with negative feelings and adverse events [30]. Several studies have found that emotional eating is linked to greater caloric intake and poor food choices, as well as higher degrees of guilt and psychopathological symptoms [9,27]. Here, we described the link between emotional eating and symptoms of anxiety in patients with obesity. Among study participants with symptoms of anxiety, we observed significantly higher levels of emotional eating than those categorized without symptoms ofanxiety. Interestingly, there were no differences in eating behaviors between patients with and without co-morbid symptoms of depression in this study.

Uncontrolled eating is defined as the tendency to overeat due to a loss of control over one's intake and subjective hunger sensations [15]. Higher disinhibition is strongly associated with greater adult weight gain, higher BMI, less success with weight reduction, and binge eating patterns [31]. Furthermore, uncontrolled eating is also associated with food cravings which contribute to episodes of overeating [32]. Finally, the relationship between (leave as was sorry) attachment anxiety symptoms and BMI was mediated by uncontrolled eating [33]. Here, we found that those with higher symptoms of anxiety also tended to report higher levels of uncontrolled eating. This is consistent with findings linking anxiety to impulsive types of food consumption, such as binge eating episodes [34].

Cognitive restraint describes the effort to restrict food intake to lose weight or avoid gaining weight [35]. Cognitive restraint is most seen in patients with obesity and individuals who go on diets. When appropriately used, cognitive restraint allows dieters to control their food intake. Previous research on personality traits and anxiety has found that greater anxiety levels in neuroticism and perfectionism are linked to effective attempts to reduce food intake. However, this does not necessarily result in weight loss in adults with obesity [36]. Our study points to the association of symptoms of anxiety with lower rates of cognitive restraint.

Previous research has looked at the relationship between self-efficacy and anxiety symptoms, demonstrating that low levels of self-efficacy are generally accompanied by high levels of anxiety [37,38]. Here, we indicate that this relationship is also found in patients with obesity. Self-efficacy is a cognitive characteristic that plays a mediating function in eating behaviors. In addition to social learning theory, other health behavior models, such as the transtheoretical model of behavior change and the health belief model, have incorporated self-efficacy as a contributor to health behavior change. For example, self-efficacy has been shown to influence the stage of change [39], enhancing positive expectancies for engaging in health behaviors [40].

Multiple factors influence eating behavior in patients with and without obesity. Given the association between eating behavior and obesity [41] and the importance of self-regulation on weight loss outcomes [42], it is essential to identify patients with anxiety-related eating behaviors, which can contribute to the pathogenesis of obesity. Identifying these patients can help us understand the pathophysiologic processes underlying obesity and hence develop individualized strategies for better weight loss outcomes and quality of life [43,44]. Our findings confirm previously published results showing the impact of anxiety on emotional eating in healthy weight patients and investigate the link between anxiety and self-confidence in resisting the impulse to eat in high risk eating situations [8,9,45]. Here, we highlight the utility of the HADS-A questionnaire to demonstrate that when compared to patients without symptoms ofanxiety, those with symptoms of anxiety exhibited higher levels of emotional eating and lower levels of cognitive restraint on the TFEQ-R21, and lower self-confidence to resist the desire to eat in five situations factors on the WEL.

For anxiety and depression, SSRIs are the first-line therapy. Our study found that the usage of SSRIs was similar in individuals with and without anxiety symptoms. One side effect of their use is body weight gain during short- and long-term management. In fact, after three months of medication therapy, individuals taking an SSRI gained 4.2 kg more than non-user of an SSRI [46]. Furthermore, after 2.5 years of SSRI medication, an increase of 2.5% of the starting body weight was discovered [47]. Current research suggests that histamine and serotonin off-target appetite-promoting pathways may have a role in weight gain [48]. Given the lack of research examing a possible association between symptoms of anxiety and weight gain, the effect of managing anxiety symptoms on eating behaviors, is unknown. More research is needed to thoroughly understand the influence of antidepressants on eating patterns in patients with symptoms anxiety.

There are several important limitations to the current study. To begin, the choice of a cross-sectional design precludes us from concluding causation. Future research with longitudinal designs is needed better to understand the relationship between different eating behaviors and symptoms of anxiety. All data were gathered via self-reported questionnaires, which might be biased owing to participants' under- or over-reporting. The inclusion of a structured diagnostic psychiatric interview would strengthen the study design by providing a diagnosis of anxiety rather than just symptoms of anxiety suggestive of an anxiety disorder. This study used a questionnaire that assess symptoms of depression and anxiety, perhaps a questionnaire that measures only symptoms of anxiety, such as the Generalized Anxiety Disorder Screener (GAD-7) would yield different results. Additionally, the sample consisted of primarily White upper-middle-class females, so how these findings apply to more diverse populations is unknown. In general, obesity and eating behavior studies have disproportionately recruited females; hence this study is skewed toward a predominantly female sample. Finally, individuals with depression who were not taking SSRIs were excluded, limiting the capacity to examine the cumulative influence of depressive symptoms on eating behaviors.

## 5 Conclusion

This study found associations between symptoms of anxiety, eating behaviors, and self-efficacy for eating. Patients with obesity with symptoms of anxiety showed higher mean scores for emotional eating and uncontrolled eating and low self-efficacy for resisting eating in a challenging situations. Given its links to negative emotions and eating behavior, employing a simple questionnaire to assess for symptoms of anxiety has the potential to be a measure included in the screening and management of obesity. Future research should look at how different components of eating behaviors alter following weight loss programs, and if incorporating anxiety management strategies into a weight loss program can improve treatment outcomes in patients with obesity and symptoms of anxiety.

## Disclosures

Dr. Acosta is a stockholder in Gila Therapeutics, Phenomix Sciences; he serves as a consultant for Rhythm Pharmaceuticals, General Mills. The authors declare that the research was conducted without any commercial or financial relationships that could be construed as a potential conflict of interest.

## Author contribution

LC, AA, and MM co-conceptualized and co-designed the study, drafted the initial data, and critically reviewed the manuscript. AC, MLR, and JS coordinated and supervised data collection, and critically reviewed the manuscript. MDH, SK, and DH provided critical feedback on the study design and critically reviewed the manuscript. All authors approved the final manuscript as submitted and agree to be accountable for all aspects of the work.

## Ethics approval and consent to participate

All procedures performed in studies involving human participants were in accordance with the ethical standards of the institutional and/or national research committee and with the 1964 Helsinki declaration and its later amendments or comparable ethical standards. For this type of study, formal consent is not required.

## Funding

Dr. Acosta is supported by NIH (NIH K23-DK114460, C-Sig P30DK84567), ANMS Career Development Award, 10.13039/100014535Mayo Clinic Center for Individualized Medicine – Gerstner Career Development Award.

## Data availability

The raw data supporting the conclusions of this manuscript will be made available by the authors, without undue reservation, to any qualified researcher.

## Declaration of competing interestCOI

The authors declare that the research was conducted without any commercial or financial relationships that could be construed as a potential conflict of interest.
